# Structured Reporting in Neuroradiology: Intracranial Tumors

**DOI:** 10.3389/fneur.2018.00032

**Published:** 2018-02-06

**Authors:** Andrea Bink, Jan Benner, Julia Reinhardt, Anthony De Vere-Tyndall, Bram Stieltjes, Nicolin Hainc, Christoph Stippich

**Affiliations:** ^1^Division of Diagnostic and Interventional Neuroradiology, Department of Radiology, University Hospital Basel, University of Basel, Basel, Switzerland

**Keywords:** structured reporting, neuroradiology, quantitative data, MRI, intracranial, tumor

## Abstract

**Purpose:**

The aim of this pilot study was to assess the clinical feasibility, diagnostic yield, advantages, and disadvantages of structured reporting for routine MRI-reading in patients with primary diagnosis of intracranial tumors as compared to traditional neuroradiological free text reporting.

**Methods:**

A structured MRI reporting template was developed covering pathological, anatomical, and functional aspects in an itemized fashion. Retrospectively, 60 consecutive patients with first diagnosis of an intracranial tumor were selected from the radiology information system/PACS system. Structured reporting was performed by a senior neuroradiologist, blinded to clinical and radiological data. Reporting times were measured per patient. The diagnostic content was compared to free text reporting which was independently performed on the same MRI exams by two other neuroradiologists. The comparisons were categorized per item as: “congruent,” “partially congruent,” “incongruent,” or “not mentioned in free-style report.”

**Results:**

Tumor-related items: congruent findings were found for all items (17/17) with congruence rates ranging between 98 and 39% per item. Four items achieved congruence rates ≥90%, 5 items >80%, and 9 items ≥70%. Partially congruent findings were found for all items in up to 50% per item. Incongruent findings were present in 7/17 items in up to 5% per item. Free text reports did not mention 12 of 17 items (range 7–43% per item). Non-tumor-related items, including brain atrophy, microangiopathy, vascular pathologies, and various extracranial pathologies, which were not mentioned in free-text reports between 18 and 85% per item. Mean reporting time for structured reporting was 7:49 min (3:12–17:06 min).

**Conclusion:**

First results showed that expert structured reporting ensured reliable detection of all relevant brain pathologies along with reproducible documentation of all predefined diagnostic items, which was not always the case for free text reporting. A mean reporting time of 8 min per patient seems clinically feasible.

## Introduction

Free text reporting is the current standard in reading neuroradiological exams. The content and the quality of the reports are heavily dependent on the radiologists’ individual training and experience. Usually, there are no predefined diagnostic items that ensure a complete assessment of a neuroimaging examination. Established classification or scoring schemes are not always applied and quantification of imaging findings is often lacking or vague. Consequently, the interreader variability can be substantial and the reproducibility of diagnostic measures may be limited. This is of special relevance when it comes to follow-up readings on the natural course of various brain pathologies or to monitoring effects of different treatments. Here, more standardization and quantification is warranted. Furthermore, recent technological advancements provide new opportunities to exploit huge data sets to create robust reference data for targeted individualized medicine (1, 2). Key is the generation of structured, reproducible, and quantitative data for large data bases. Structured reporting may be an easy to implement, yet imperfect initial step in this direction by, e.g., facilitating data extraction for big data analysis.

In this study, we assessed a structured reporting routine designed for neuroradiological routine reading of brain MRI in patients first diagnosed for intracranial tumors.

In the last decade, several studies have been performed to examine the usefulness of structured radiological reporting in comparison to free-style reporting. It was found that the referring clinicians were more satisfied with structured reports than with free-style reports (3). A reduction in omissions of findings was detected (4). Radiological societies like the RSNA have put effort into developing structured reporting templates (5). While structured reports are accepted for certain body regions, like breast or prostate, structured reporting in other fields is not widely used, e.g., in neuroradiology. Although RSNA provides a general template for reading brain MRI (6) there are no templates available dedicated to specific brain pathologies such as intracranial tumors.

In intracranial tumor reporting, many items have to be considered and accurately reported, e.g., exact anatomical location, size, number of lesions, dissemination, contrast enhancement, type of enhancement, involvement of so-called eloquent areas, edema, space-occupying effect, bleeding, signal changes in diffusion weighted imaging (DWI), and perfusion characteristics. In clinical routine, it is considered beneficial to perform complete and thorough reporting in time-efficient manner: to this end, we developed a structured reporting template for initial intracranial tumor reports which was evaluated in 60 patients with intracranial tumors.

The first aim of this pilot study was to assess the clinical feasibility and find out if structured reporting yields more complete diagnostic information in comparison to conventional free-style reporting. Second, we aimed to detect and describe weaknesses of such structured reporting and finally, we analyzed our procedure in order to improve both template and reporting process taking measured reporting times into account.

## Materials and Methods

This descriptive feasibility pilot study was approved by the institutional review board (EKNZ BASEC 2016-00167). The ethical committee waived the requirement for written informed consent.

A radiology information system (RIS, Centricity RIS-i5.0, General Electric Company, 2015) search query for the MRI protocols used in our institution for suspicion of primary intracranial tumors was performed, and 60 patients with reported primary tumor and no previous reports were randomly drawn from a collective ranging from 2013 to 2016.

The patient data sets were exported to the Syngovia platform (Siemens Healthineers, Siemens Healthcare GmbH, 2009–2017). The evaluation was conducted using a standardized template which was developed by two neuroradiologists each with more than 14 years reading experience in neuroradiology alone (Christoph Stippich, Andrea Bink) and which was finally completed and approved by Christoph Stippich. The structured reporting was done by a senior neuroradiologist (Andrea Bink, 15 years reading experience in Neuroradiology) who did not conduct the previous neuroradiological reporting and who was blinded to patients’ clinical information and the written reports. Times required for structured reporting were documented for each patient separately.

After completion of the structured reporting process a comparison to the medical content of the free-style reports documented in RIS was performed by the senior neuroradiologist Andrea Bink. These results were documented in an MS-Excel Sheet (Microsoft Excel 2010) with anonymized patient data. Findings were categorized by “congruent,” “partially congruent,” “incongruent,” and “not mentioned in free-style report.”

17 predefined tumor-related and 4 non-tumor-related items are summarized in Table 1.

**Table 1:** Item definitions.

	Item content
	Report on
**Item**	
Number	Lesion count up to five supratentorial and up to five infratentorial
Location^a^	Involved anatomic structures: lobes, gyri, corpus callosum, basal ganglia, thalami, ventricles, ependyma, meninges, brain stem, cerebellum, vermis, sella, cranial nerves, skull base, upper cervical spine
Eloquence	Involvement of motor cortex, pyramidal tracts, sensory cortex, Broca’s area, Wernicke’area, visual pathway, auditory pathway, basal ganglia, thalami, hypothalami, brain stem, dentate nuclei^b^
Diameter 1, 2, 3	Three different diameters taken regardless of which plane or angulation of the contrast-enhancing tumors
2D size ceT1w	Two diameters measured in the largest tumor diameter perpendicular to each other on axial plane
2D size ceT1w hgg^c^	Only high-grade gliomas: two diameters measured in the largest tumor diameter perpendicular to each other on axial plane
2D tumor size T2w/fluid-attenuated inversion recovery (FLAIR)	Two diameters measured in the largest FLAIR/T2w signal changes perpendicular to each other on axial plane
Edema^d^	Maximum perifocal diameter of edema with grading: small (≤1 cm), moderate (≤3 cm), or extensive (≥3 cm)
Space^e^	CSF circulation problems, midline-shift, compression of: ventricles, basal cisterns, herniation: transfalxial, transtentorial, uncal, transforaminal
Blood^f^	Susceptibility weighted imaging signal changes and their space-occupying effect
DWI^g^	Diffusion changes: categorized as facilitated, restricted, or mixed
Perfusion	Hyperperfused areas in the brain
Vessels	Vessel pathologies, e.g., stenosis, aneurysms
White matter	Description of signs of microangiopathy under consideration of Fazekas^h^ classification
Brain volume	Description of loss of brain volume
Viscerocranium	Description of pathologies in orbits, paranasal sinuses, mastoids and any other part of included viscerocranium

*^a^Location: location subdivided into supratentorial intracerebral, supratentorial extracerebral, infratentorial intracerebral, infratentorial extracerebral*.

*^b^Modified from Sawaya et al. (7)*.

*^c^High-grade gliomas*.

*^d^Edema: size of maximum perifocal edema*.

*^e^Space: space-occupying effect*.

*^f^Blood: signal changes indicating hemorrhage*.

*^g^DWI, diffusion weighted imaging; DWI and apparent diffusion coefficient maps taken into account*.

*^h^Fazekas: Fazekas et al. (8)*.

The free-style reports were performed by one radiologist in training and one consultant neuroradiologist who was finally responsible for signing the report (reading experience between 10 and 18 years in neuroradiology).

### MRI Protocols

All MRI studies were performed at 3T (Siemens, Magnetom Skyra, Siemens Healthineers, Erlangen, Germany) using a commercially available 20 channel head-neck coil. The tumor MRI protocol consisted of: magnetization-prepared rapid gradient-echo sequence (MPRAGE), DWI, susceptibility weighted imaging (SWI), and after contrast media application, DSC-perfusion imaging, T2 TSE-weighted images, fluid-attenuated inversion recovery (FLAIR), and T1 MPRAGE post contrast images. Details concerning sequence parameters: *Precontrast: MPRAGE*: TR/ms 2,300, TE/ms 2.27, TI/ms 900, FOV/mm 250, slice thickness/mm 1, matrix 256 mm × 256 mm, voxel size 1 mm × 1 mm × 1 mm, orientation sagittal, acquisition time/min 04:44, images evaluated: source, MPR: axial, coronal. *DWI* TR/ms 9,600, TE/ms 98, FOV/mm 220, slice thickness/mm 3, matrix/mm 162, voxel size 1.4 mm × 1.4 mm × 3.0 mm, orientation axial, acquisition time/min 1:38, images evaluated: axial. *SWI* TR/ms 27, TE/ms 20, FOV/mm 220, slice thickness/mm 3, matrix 256 mm × 256 mm, voxel size 0.9 mm × 0.9 mm × 3.0 mm, orientation axial, acquisition time/min 02:15, images evaluated: axial. *Post contrast: DSC-Perfusion* TR/ms 1,600, TE/ms 30, FOV/mm 230, slice thickness/mm 6, matrix 128 mm × 128 mm, voxel size 1.8 mm × 1.8 mm × 6.0 mm, orientation axial, acquisition time/min 01:34, images evaluated: axial.*T2 TSE* TR/ms 4,070, TE/ms 89, FOV/mm 200, slice thickness/mm 3, matrix 384 mm × 384 mm, voxel size 0.5 mm × 0.5 mm × 3.0 mm, orientation coronal, acquisition time/min 03:01, images evaluated: coronal. *FLAIR* TR/ms 9,000, TE/ms 81, TI/ms 2,500, FOV/mm 220, slice thickness/mm 3, matrix 320 mm × 320 mm, voxel size 0.7 mm × 0.7 mm × 3.0 mm, orientation axial, acquisition time/min 02:44, images evaluated: axial. *MPRAGE* parameters: see above precontrast MPRAGE.

### Definitions for Evaluation of Tumor-Related and Non-Tumor-Related Items

An overview of the evaluation of items is given in Table 2.

**Table 2:** Item evaluation.

	Definitions of categories for comparison between free-style and structure reports			
	Congruent	Partially congruent	Incongruent	Not mentioned
**Item**				
Number	The same findings in free-style and structured reports	Different number count	Supratentorial lesions described and infratentorial lesions missed or *vice versa*	No comment in free-style report

Location		Locations differed in part, e.g., a tumor extended from right frontal to parietal in one report while in the other report it was only right frontal	Locations differed completely or one location was not mentioned in the structured report which was mentioned in free style^a^	

Eloquence		At least one eloquent area differed	Eloquent areas differed completely	

Diameter		Not all tumors measured	No reproducible measurements	

2D size ceT1w		Correct measurement, but not all tumors measured	No reproducible or not perpendicular measurements	

2D size ceT1w hgg		Correct measurement, but not all tumors measured	No reproducible or not perpendicular measurements	

2D size T2w/fluid-attenuated inversion recovery		Not all lesions measured or only one dimension measured	No reproducible measurement	

Edema		Similar measurements or judgments, but not for all lesions	Different judgments or measurements for all lesions	

Space		Same statement concerning CSF circulation problems but not the same space-occupying signs described	One report mentions CSF circulation problems while the other describes no problems	

Blood		Susceptibility weighted imaging (SWI) signal changes in both reports, but only one report describes local space-occupying effect	SWI signal changes in one report while the other describes none	

Diffusion weighted imaging		Not all lesions described in the same manner	Divergent statements, e.g., restricted diffusion versus no diffusion changes	

Perfusion		Not all lesions described in the same manner	Divergent statements, e.g., hyperperfusion versus no hyperperfusion	

Vessels		At least one pathologic finding different between the reports	No abnormality versus abnormality	

White matter		One report mentions microangiopathy Fazekas 1, the other microangiopathy Fazekas 2	No microangiopathy vs. microangiopathy or microangiopathy Fazekas 1 in one report vs. microangiopathy Fazekas 3 in the other report	

Brain volume		At least one pathologic finding different between reports, e.g., general volume loss with focal special atrophy in one report versus only general volume loss in the other report	Divergent statements of both reports, e.g., no volume loss versus volume loss	

Viscerocranium		At least one pathologic finding different between the reports	Divergent statements of both reports, e.g., no abnormality versus opacification of maxillar sinus	

*^a^Non-mentioning of a tumor location in the structured report was considered a severe mistake and not only an omission issue*.

### Ranking of Items

In order to describe which items achieved the best accordance we ordered the items from highest to lowest congruence rates. In case of equal congruence percentages, the items were ranked according to the highest percentage of partially congruent findings.

### Time Measurements for Structured Reporting

**Figure 1:**
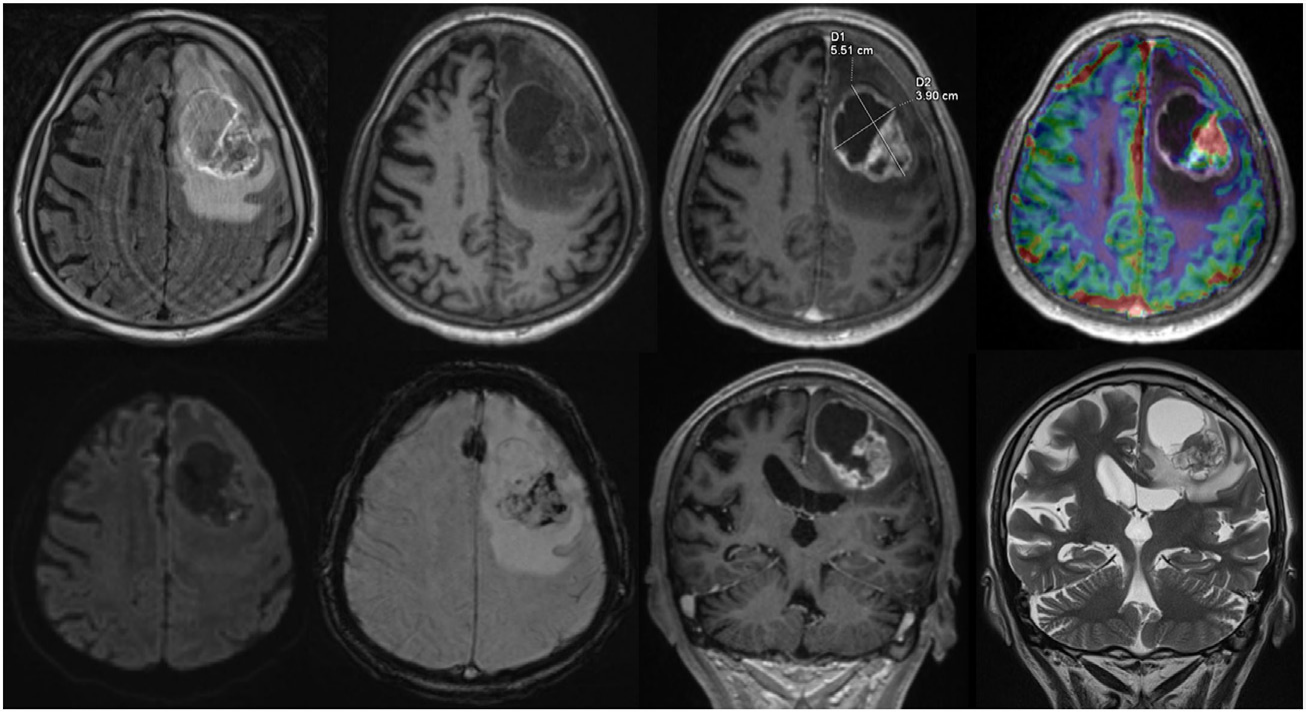
Screen view Structured reporting with 2D size measurements, upper row, axial: fluid-attenuated inversion recovery, native T1w, ceT1w, DSC-Perfusion, lower row, axial: diffusion weighted imaging, susceptibility weighted imaging, coronal: ceT1w, T2w.

For each structured report the reporting time was measured. Reporting time was defined from the start of reading the first diagnostic sequence until the finalization of filling out the template form. An example of a screen during reading is given in Figure 1.

Identical mounting of all MR-studies was performed prior to reading on the Syngovia platform and procedural remarks were documented, when relevant. The time needed for these tasks was not included in the measurements.

## Results

The 60 MRI exams included were performed between 2013 and 2016 (40 in 2016, 13 in 2015, 4 in 2014, 3 in 2013). All patients were examined with the identical MRI tumor protocol. Additional SWI was available in 54 patients and perfusion measurements in 38 patients.

The radiological impressions of the tumors which were common in free-style and structured reports were as follows: metastasis (*n* = 16), glioblastoma (*n* = 10), meningeoma (*n* = 8), vestibular schwannoma (*n* = 7), oligodendroglioma (*n* = 4), lymphoma (*n* = 4), low-grade glioma (*n* = 3), all other high-grade gliomas (*n* = 3), DNET (*n* = 3), ganglioglioma (*n* = 2), pleomorphic xanthastrocytoma (*n* = 1), and subependymoma (*n* = 1). Two vestibular schwannomas were described in the free-style reports in patients with metastasis. Only one of these two vestibular schwannomas was described in the structured report.

**Figure 2:**
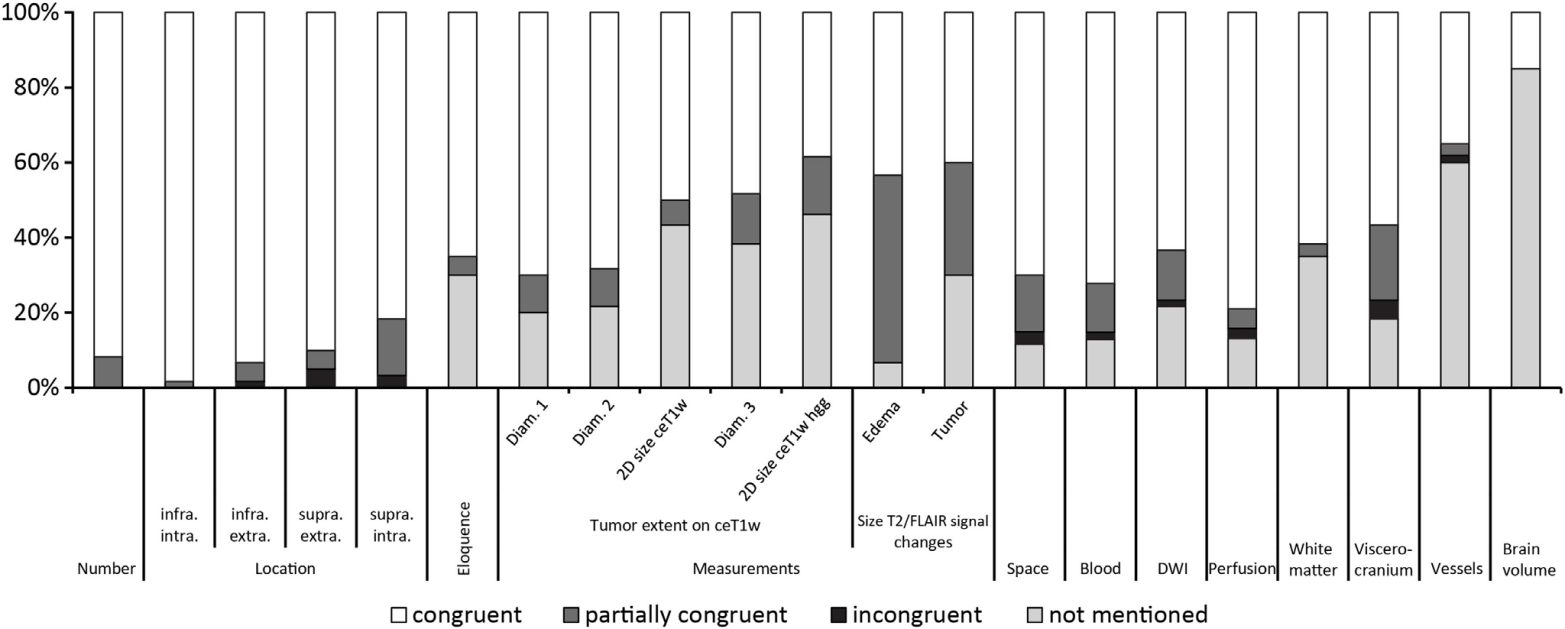
Results ordered by topic and within the topics location, measurements and T2w/fluid-attenuated inversion recovery signal changes by congruence.

Figure 2 gives an overview of the results for all 21 items.

### Tumor-Related Items

Table 3 summarizes the results of the comparison between structured and free-style reported findings for tumor-related items. Congruent findings were found from 39 to 98% in the 17 items. Partially congruent findings were found in 17 of 17 items in up to 50%. Incongruent findings were seen in 7 of the 17 items in up to 5%. In 12 items free text reports did not mention the finding (range, 7–46%).

**Table 3:** Results of tumor-related items.

	Number	Location				Eloquence	Measurements										
		infra. intra.	infra. extra.	supra. extra.	supra. intra.		Tumor extent on ceT1w					Size T2/fluid-attenuated inversion recovery signal changes		Space	Blood	Diffusion weighted imaging	Perfusion
	
							Diam. 1	Diam. 2	2D size ceT1w	Diam. 3	2D size ceT1w hgg	Edema	Tumor				
No. patients incl.	60	60	60	60	60	60	60	60	60	60	13	60	60	60	54	60	38
Congruent	55	59	56	54	49	39	42	41	30	29	5	26	24	42	39	38	30
Partially congruent	5	1	3	3	9	3	6	6	4	8	2	30	18	9	7	8	2
Incongruent	0	0	1	3	2	0	0	0	0	0	0	0	0	2	1	1	1
Not mentioned	0	0	0	0	0	18	12	13	26	23	6	4	18	7	7	13	5
**In %**																	
Congruent	92	98	93	90	82	65	70	68	50	48	39	43	40	70	72	63	79
Partially congruent	8	2	5	5	15	5	10	10	7	13	15	50	30	15	13	13	5
Incongruent	0	0	2	5	3	0	0	0	0	0	0	0	0	3	2	2	3
Not mentioned	0	0	0	0	0	30	20	22	43	39	46	7	30	12	13	22	13

#### Number of Lesions

The numbers of lesions found was in good accordance between structured reporting and free-style reporting (Table 3). All five patients with “partially congruent” ratings had brain metastasis. One of these patients had one cerebellar metastasis on the right and a vestibular schwannoma on the left. The vestibular schwannoma (size 4.5 mm × 4.5 mm × 4 mm) was not documented in the structured report.

#### Location

No location was missed in the free-style reports. Six incongruent ratings between the reports were found: Two concerning supratentorial intracerebral, three involving the supratentorial extracerebral and one the infratentorial extracerebral location, the latter being the vestibular schwannoma not described in the structured report. The majority of “partially congruent” findings were found in nine patients with supratentorial intracerebral lesions: Three patients with metastasis, two with lymphoma, one glioblastoma, one malignant astrocytoma, and two with oligodendrogliomas.

#### Eloquence

30% of free-style reports did not explicitly mention if an eloquent location was involved or not involved. Partial differences between the reports existed in 5%.

#### Tumor Diameter

Tumor size was mentioned in different fashions in free-style reports, e.g., most of the time one diameter, second most two diameters, and least three diameters were measured. Single diameter measurement was found mainly in patients with multiple lesions. 20% of free-style reports did not mention a measurement.

#### 2D Size of Tumors on T1w Post Contrast Images

We used for 2D size assessment the measurement technique of two largest tumor diameters perpendicular to each other on axial post contrast T1-weighted images for our primary tumor assessment, inspired by the Macdonald part of RANO (Response Assessment in Neuro-Oncology) (9, 10). 2D size was not mentioned explicitly in the reports but could be reproduced by the saved measurements in the PACS system as underlying source of reporting in 50% of reports. This means that a relevant number of measurements was conducted, but not documented.

#### 2D Size of High-Grade Gliomas on T1w Post Contrast Images

The patients with differential diagnosis of malignant glioma were additionally scrutinized. In 10 glioblastoma and 3 high-grade gliomas only 39% of the free-style reports were congruent to the structured report, in 15% only a partial congruence existed and 46% free-style reports did not measure 2D size.

#### 2D Measurement of Tumor-Induced T2w/FLAIR Signal Changes

30% of free-style reports did not mention any size of the tumor-related T2 or FLAIR signal changes, only partial information was given in 30% of free-style, and similar results were generated in 40% of free-style and structured reports.

#### Size of Maximum Perifocal Edema

The comparison showed that 43% of free-style and structured reports generated congruent results, 50% were partially congruent and in 7% of free-style reports maximum perifocal edema size was not mentioned.

#### Space-Occupying Effect

The comparison showed that 70% of free-style and structured reports generated congruent results, 15% were partially conguent and 12% of free-style reports did not comment on space-occupying effect. Free-style and structured reports generated incongruent findings in 3%; in the two cases the free-style report gave space-occupying remarks but did not explicitly state that there was a CSF circulation problem.

#### Signal Changes Indicating Intratumoral Bleeding

Susceptibility weighted imaging was performed in 54 patients. 72% of the reports were concordant concerning the finding “intratumoral SWI signal hypointensities,” this was not mentioned in 13% of free-style reports. 2% incongruent and 13% partially congruent findings between the free-style and structured reports were detected. In the patient with the incongruent report the free-style report stated no intratumoral bleeding while the structured report did.

#### Signal Changes on DWI

63% of free-style and structured reports showed the same results concerning signal changes in DWI whereas 13% showed only partial congruence. 22% of free style reports did not mention DWI in the report. In one patient, an incongruent finding was seen due to a missed small embolic infarction in the white supratentorial matter in the structured report.

#### Tumor-Associated Perfusion Changes

DSC-perfusion imaging was performed in 38 patients. Free-style and structured reports showed 79% congruent results, 5% partially congruent results, and 3% (one patient) incongruent results. 13% of free-style reports did not mention—although performed—perfusion results. The patient with incongruent reports had a description of hyperperfused intratumoral areas in free-style reporting and a statement of no hyperperfusion in the structured report.

### Non-Tumor-Related Items

The results of the comparison between free-style and structured reports’ findings concerning the non-tumor-related items can be seen in Table 4.

**Table 4:** Results of non-tumor-related items.

	Other items			
	White matter	Viscerocranium	Vessels	Brain volume
No. patients incl.	60	60	60	60
Congruent	37	34	21	9
Partially congruent	2	12	2	0
Incongruent	0	3	1	0
Not mentioned	21	11	36	51
**In %**				
Congruent	62	57	35	15
Partially congruent	3	20	3	0
Incongruent	0	5	2	0
Not mentioned	35	18	60	85

Congruent findings were found between 15 and 62% in the four items. Partially congruent findings were found in 3 of 4 items ranging between 3 and 20%. Incongruent findings were seen in 1 of the 4 items in 5% of reports. In all four items free-text reports did not mention the addressed topic (range, 18–85%).

#### White Matter

Congruent findings were seen in 62%, partially congruent findings in 3% and in 35% no statement about microangiopathy was found in free-style reports.

#### Viscerocranium

Congruent findings were found in 57%, partially congruent in 20% and incongruent in 5% of reports. Findings of the viscerocranium were not mentioned in 18% of free-style reports. The three incongruencies resulted from free-style reports denying mucosal swelling of the paranasal sinuses whereas structured report stated mucosal swelling.

#### Vessels

The comparison showed that 35% of free-style and structured reports generated congruent results, 3% were partially congruent, 2% were incongruent and the topic “vessels” was not mentioned in 60% of free-style reports. The incongruent finding was due to a missed arteria communicans anterior aneurysm in the structured report.

#### Brain Volume

85% of free-style reports did not address brain volume. Congruent findings between structured and free style were detected in 15% of reports.

### Items Orded by Rank

**Figure 3:**
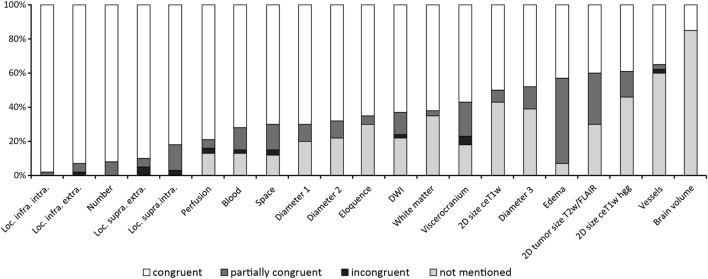
Results ordered by rank (highest to lowest percentages in the category “congruent”).

Figure 3 shows the results of the ranking of items.

### Time per Report

The mean time for structured reporting was 7 min and 49 s (median, 07:09 min; range, 3:12–17:06 min).

### Findings Not Reported in the Structured Report

The structured reporting missed a vestibular schwannoma, an aneurysm of the anterior communicating artery and an embolic small infarction in the white matter.

The texture of tumor was not explicitly addressed in the structured report and in general it was clearer by reading the free-style reports as necrosis, cysts, and solid areas were described.

## Discussion

In this pilot feasibility study, structured reporting in patients with first diagnosis of an intracranial tumor could be performed on average in about 8 min and seems feasible for clinical routine neuroradiology service. When compared to established free-style reporting, structured reporting ensured a more thorough detection and description of tumor-related items and even more non-tumor-related clinically relevant findings. While 7–46% of free-style reports failed to address 12 of 17 tumor-related items and 18–85% failed to address all four non-tumor-related items, structured reports missed only three pathologies, i.e., a vestibular schwannoma, an aneurysm of the anterior communicating artery and a small embolic infarction in the white matter. Interestingly, the expert reader did not document these findings in the structured report although it asked for tumors, vessels, and DWI restrictions. This implies that structured reporting cannot fully exclude all individual reading errors, but helps to reduce incompleteness of neuroradiological reports to a minimum. In this context, the subjective observation of the reporting radiologist was, that using a structured template also modifies the way the radiological exams are looked at. A “checklist” needs to be completed as an additional intellectual task, which may interfere with the individual “reading routine” each experienced neuroradiologist has developed. This may lead to the effect that with completion of the list the task is done and secondary findings are not checked for with enough effort. Although the template led to reduced omission rate in reporting, the radiological method of analyzing all structures without secondary influence has additional value and by no means can experience and knowledge of a radiologist be replaced completely by structured reports or artificial intelligence in their current form.

### Tumor-Related Items

Numbers of tumors and their locations were the items with the highest percentages of congruent findings between free-style and structured reports (range, 82–98%) and these were the only five items without missing information in free style. Between 70 and <80% congruent findings were seen in the item “perfusion,” “SWI signal changes indicating bleeding,”, “tumor diameter 1,” and “space-occupying effect.” Interestingly, “perfusion” (79%) reached a higher percentage of congruent findings than DWI (63%) and a second tumor diameter was found to be congruent only in 68%. Generally speaking, a basic tumor report can be performed by addressing all of the above mentioned items. But some remarks need to be made: Free-style reports did not address all these items. In 12% (“space-occupying effect”), 13% (“perfusion” and “SWI signal changes indicating bleeding”), 20% (“tumor diameter 1”), and 22% (“tumor diameter 2” and “DWI”) of free-style reports remarks were missing. Although in our patient collective no acutely dangerous findings were overlooked, the completeness and information provided by free-style reporting was substantially lower. Taking into account that free-style reports did not address the topic “eloquence” in 30%, the quality of reports becomes, yet again, an issue. With structured reporting important tumor information can not be as easily “overlooked,” as the items need to be answered on the template. On the other hand, if the template is not designed to cover all relevant imaging features, such as intratumoral necrosis, cysts, or solid areas, this information will not be provided at all. This underlines the importance of performing a feasibility-check for newly designed templates (like in this study) for structured radiological reporting. We will add these missing but relevant items to our template. In addition, the information provided to our referring physicians will be enhanced by implementing representative images into the written neuroradiological reports.

The ranking of the items as documented in Figure 3 shows the method of traditional reading quite well as the first 13 items seem to represent “normal” standard reporting which necessarily does not include measurement of edema or measurement of a third diameter of lesions.

Measurement of tumors is established for follow-up assessment of high-grade gliomas by the RANO criteria (10). In this study, we assessed images with first tumor diagnosis for diverse tumor entities. We looked at different methods of measurement. We considered one diameter measurement in three planes as represented by “tumor diameter 1–3” (here the angle between the diameters could be random) as well as tumor measurement by two diameters in one axial plane where the diameters were perpendicular to each other. We evaluated the 13 patients with high-grade gliomas separately. The measurement items had in general the highest rates of omissions in free-style reports, increasing from “tumor diameter 1” (20%) up to “2D size of high-grade gliomas on T1w post contrast images” (46%). As two diameters are regularly measured a third diameter is performed in less than 2/3 of the cases. A possible explanation could be—while RANO is referring to measurements in the axial plane in the follow-up situation—that no primary preoperatively established measurement system exists.

Except for the 2D size measurement of high-grade gliomas T2w/FLAIR signal changes achieved the lowest congruent results in tumor-related items, i.e., for “2D size of tumor-induced T2w/FLAIR signal changes” 40% and “size of maximum perifocal edema” 43%. Congruent results were found in “2D size of tumor-induced T2w/FLAIR signal changes” when the tumor was identified by its signal changes on T2w/FLAIR and showed no enhancement on T1w post contrast images and was, therefore, measured on T2w or FLAIR images or in the case when the tumor had, incidentally, the same size on T1w post contrast as on T2w/FLAIR images. The perifocal maximum edema size was the item which had the most partially congruent reports (50%) which was mainly due to the strict definition accepting only similar measurement or classification in either minor (≤1 cm), moderate (≤3 cm), or extensive (≥3 cm) and evaluation of all lesions (up to 10) as congruent.

### Non-Tumor-Related Items

In the free-style tumor reports vessels, white matter (microangiopathy), brain volume, and viscerocranium received a minor priority shown by the percentages of omissions (“vessels” 60%, “white matter” 35%, “brain volume” 85%, and “viscerocranium” 18%). Interestingly, findings of the viscerocranium were least omitted. “Microangiopathy” and “viscerocranium,” the latter consisting mainly of reports on the paranasal sinuses, had the highest congruence, 62 vs. 57%, whereas “brain volume” was mostly neglected in free-style reports of intracranial tumors. The reason for this might be primarily due to the different focus while reporting an intracranial tumor setting. Furthermore, it has to be considered that due to space-occupying effects a proper report on brain volume might not be possible in some cases. In the nine patients with reports on brain volume, there was a 100% congruence between free-style and structured reports. In summary, the four items showed that, if a tumor was present, it was the main focus of the report. This is also underlined by the missing report of an anterior communicating artery aneurysm missed in the structured report showing that even if asked for alternative pathology the reader-bias can be misleading. On the other hand, it has been shown that structured reporting can help to decrease missed findings (4).

### Limitations

A manual documentation of findings was performed in the template as technical issues could not be solved during the time of our study. For implementation in the clinical routine a digital integration into the reporting system by speech recognition voice control reporting is necessary. To cite the results of a focus group meeting “Structured reporting will fail if it compromises accuracy, completeness, workflows, or cost-benefit balance” (11). In the future, a reduction of reporting time may be achieved using template integration in the electronic reporting system taking into account that the mean reporting time was already clinically feasible in the presented setting. A comparison of structured reporting times to free-style reports was not possible as completed, existing free-style reports were used in this study. This is a limitation due to the retrospective nature of this study and, therefore, we cannot provide the exact free-style reporting times for the reports of the 60 included patients. But we would like to add that the mean reporting time of the expert reader in general intracranial tumor reporting is about 10 min (measured by the reader as mean of 3 months). The free-style reports performed by radiologist in training and senior neuroradiologist take between 15 and 30 min (measured by the readers as range during 3 months).

A further limitation of this pilot study was that free-style reports would have probably performed better in mentioning more of the features of the structured reporting list in a prospective setting. Additionally, it needs to be mentioned that not all items were covered by our preliminary template. Tumor texture items like, e.g., necrosis, cysts, or solid areas were not explicitly addressed as well as spectroscopic and fMRI items. In a future template, these items will be included, e.g., for spectroscopy and fMRI in addional advanced tumor templates. Due to important diagnosis missed by structured reporting the template should be improved by adding a checklist highlighting relevant items, like, e.g., questions for infarcts or aneurysms comparable to an “autopilot” in aviation.

Within this retrospective work, it was not possible to measure consistency of 2D size measurements. It is known that inter-observer agreement on tumor boundaries in high-grade gliomas is problematic on MRI images (12). In the recent years, automatic segmentation tools have improved reproducibility of measurements of tumor metrics (13, 14). In future prospective tumor studies automatic measurements should be part of the applied evaluation tools.

Structured reporting and comparison of structured reports and free style was performed by one senior neuroradiologist who took part in the template generation. This design was chosen on purpose as we wanted to avoid possible effects, e.g., changes in time consumption, due to learning curves from non-primarily involved readers. We refrained from a multireader setting or focus on reproducibility on purpose as this study was planned as a first test-phase to discover major drawbacks of the template from an expert reader point of view. In the future, the template will be revised on the basis of this expert reader evaluation, implemented in routine reporting and will be tested by less experienced readers.

This study focused on the quality of structured and free-style reporting and did not address the impression or degree of satisfaction of the clinicians. It is known that referring clinicians judge structured reports better than free-style reports (3). This study was an initial internal quality check for structured reporting of intracranial tumors. In a second step, the template will be discussed with and revised according to the needs of our clinicians as has been done successfully previously (15). The revised templates’ reports and free-style reports will be used as a survey to explore satisfaction of the clinicians with each structured report. Furthermore, by implementing structured reports in our radiological daily routine we aim to improve report quality and simplify extraction of data for major analysis like big data evaluation (1) and through this enhance the scientific value of daily routine work.

## Conclusion

In this pilot feasibility study, structured reporting on intracranial tumors was feasible for neuroradiological routine, required roughly 8 min of reading time and provided reproducible and more complete diagnostic information as compared to traditional free-style reporting. Full embedding of voice-controlled structured reporting into the radiological reporting system may substantially improve practicality and help to further reduce the reporting times. In rare cases non-tumor-related additional findings may be overlooked due to using predefined reporting templates. A second check for not requested findings after completing the template may be helpful. Finally, after having evaluated our preliminary structured reporting data, the appropriate and complete design of structured reporting templates is crucial, should be tested in clinical routine and modified according to an interdisciplinary consensus.

## Ethics Statement

All procedures performed in this study involving human participants have been approved by the appropriate ethics committee “Ethikkommission Nordwest- und Zentralschweiz” (EKNZ BASEC 2016-00167), were in accordance with the ethical standards of the institutional and/or national research committee and with the 1964 Helsinki declaration and its later amendments.

## Author Contributions

AB and CS: substantial contributions to the conception, design of the work, and the acquisition, analysis, and interpretation of data for the work, drafting the work or revising it critically for important intellectual content, final approval of the version to be published, and agreement to be accountable for all aspects of the work in ensuring that questions related to the accuracy or integrity of any part of the work are appropriately investigated and resolved. JB, JR, AV-T, BS, and NH: acquisition, analysis and interpretation of data for the work, drafting the work or revising it critically for important intellectual content, final approval of the version to be published, and agreement to be accountable for all aspects of the work in ensuring that questions related to the accuracy or integrity of any part of the work are appropriately investigated and resolved.

## Conflict of Interest Statement

The Department of Radiology, University Hospital Basel, Switzerland receives financial support from Bayer Healthcare, Bracco and Guerbet and has a research agreement with Siemens Healthineers. The submitted work is not related to these agreements. CS receives no other financial support related to the submitted work. The other authors declare no conflict of interest. The content of the presented work is not influenced by any financial support.
